# Extended Superolateral Periosteal Flap for Single-Stage Total Lower Eyelid Reconstruction

**DOI:** 10.1055/a-2908-5494

**Published:** 2026-08-21

**Authors:** Pattana Ongkasuwan

**Affiliations:** Division of Plastic and Reconstructive SurgeryDepartment of General Surgery 68012 Nakornping HospitalDon KaewChiang MaiThailand

**Keywords:** eyelid, eyelid neoplasm, carcinoma, basosquamous, surgical flaps, plastic surgery procedures, periosteum

## Abstract

Total lower eyelid reconstruction requires restoration of both lamellae and prevention of postoperative malposition. Traditional two-stage approaches often require a prolonged period of monocular vision. This report describes an extended superolateral periosteal flap harvested along the superior orbital rim to within 2-3 mm of the supraorbital notch, achieving a 35-mm length for single-stage total lower eyelid reconstruction. A 71-year-old woman with basosquamous carcinoma involving the entire lower eyelid underwent excision with 5-mm margins, creating a 30 × 35 mm defect. The periosteal flap was secured to the medial canthal tendon, and the conjunctiva was advanced to line the flap. The anterior lamella was reconstructed using a cheek rotation flap. Early follow-up demonstrated satisfactory eyelid position with transient chemosis. At 3 months, a stable margin position was maintained, and no early ectropion, entropion, symblepharon, or lagophthalmos was observed. Compared with free tarsoconjunctival grafts, a vascularized periosteal flap provides intrinsic blood supply and may support tissue survival when recipient bed vascularity is limited. This extended periosteal flap may serve as a single-stage alternative for complete lower eyelid defects while preserving binocular vision during recovery. Longer-term follow-up was limited due to the patient's death from an unrelated medical condition.

## Introduction

Total lower eyelid defects present reconstructive challenges that require simultaneous restoration of structural support, mucosal lining, and external coverage. Traditional approaches, such as the Hughes tarsoconjunctival flap or acellular dermal matrix grafts, necessitate multistaged procedures with prolonged monocular vision. 1 2 3 Periosteal flaps offer vascularized tissue for posterior lamella reconstruction without such limitations; however, the achievable flap length has restricted their application to subtotal defects. 4 5 6 This case report describes an extended superolateral periosteal flap technique that enables single-stage total lower eyelid reconstruction.

## Idea

This study adhered to the tenets of the Declaration of Helsinki. Written informed consent for publication of clinical details and photographs was obtained from the patient's next of kin. A 71-year-old woman presented with basosquamous carcinoma involving the entire lower eyelid ( Fig. 1 ). Following clinical evaluation and imaging, no evidence of nodal or distant metastases was identified. The tumor measured 25 mm in greatest dimension with full-thickness eyelid involvement, staged as T3cN0M0 (Stage III) according to the American Joint Committee on Cancer 8th edition. 7

**Fig. 1:**
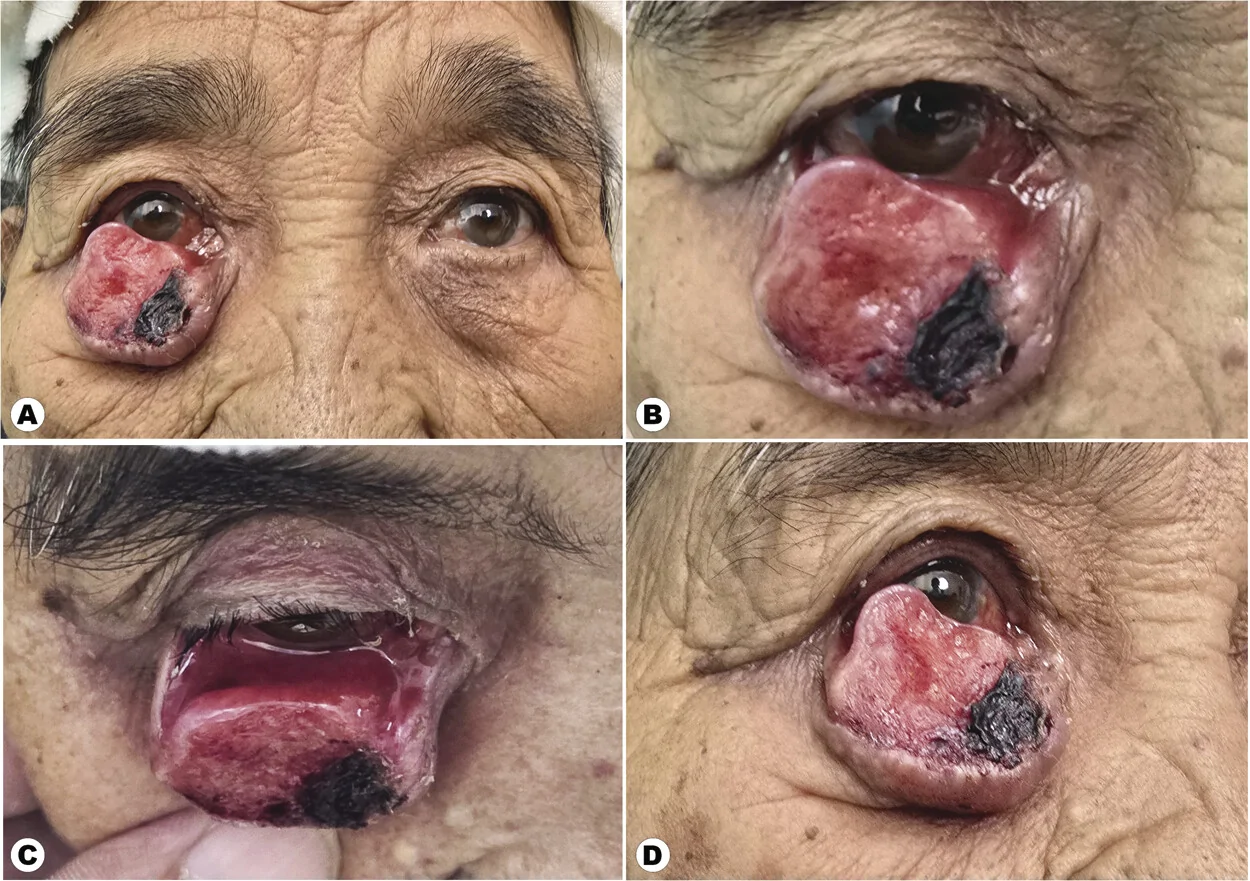
Preoperative photographs demonstrating basosquamous carcinoma with total lower eyelid involvement. ( **A** ) Frontal view with eyes open. ( **B** ) Close-up of tumor. ( **C** ) View with lower eyelid everted. ( **D** ) Oblique view showing tumor extent.

Under general anesthesia, the supraorbital notch was marked by palpation to establish a safe medial boundary for dissection. Following tumor excision with 5-mm oncologic margins, including the orbicularis muscle, the defect measured 30 × 35 mm ( Fig. 2A–D ). Surgical margins were confirmed clear by permanent histopathological examination.

**Fig. 2:**
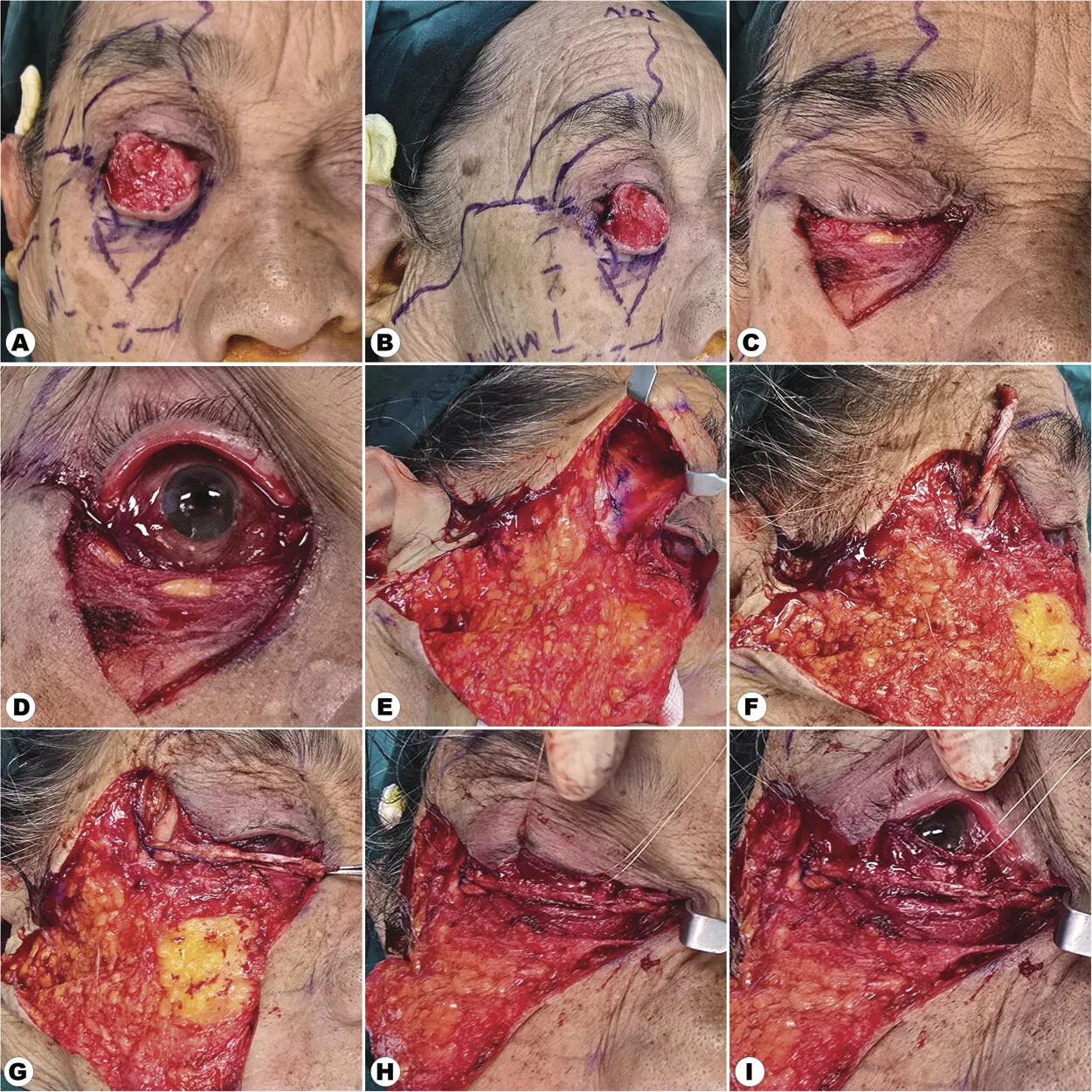
Intraoperative photographs. ( **A, B** ) Marking of the supraorbital notch and periosteal flap design along the superior orbital rim. ( **C, D** ) Defect measuring 30 × 35 mm after tumor removal. ( **E–G** ) Extended superolateral periosteal flap measuring 35 mm in length. ( **H, I** ) Medial fixation and inferior fornix conjunctival advancement.

For posterior lamella reconstruction, a periosteal flap measuring 35 mm in length and 6–8 mm in width was designed along the superior orbital rim, extending from a lateral base at the lateral canthal region, across the frontozygomatic suture, to a medial endpoint within 2 to 3 mm of the supraorbital notch ( Fig. 2E–G ). Subperiosteal elevation was performed from medial to lateral, with preservation of the lateral canthal attachment as the vascular pedicle. The flap was transposed inferiorly and secured to the medial canthal tendon remnant using 6–0 non-absorbable monofilament sutures ( Fig. 2H, I ).

The inferior fornix conjunctiva was advanced superiorly to cover the periosteal surface and secured with 5–0 absorbable sutures. For anterior lamella reconstruction, a cheek rotation cutaneous flap was elevated and advanced medially. The donor sites achieved primary closure with 4–0 absorbable sutures ( Fig. 3 ). A schematic illustration of the complete surgical sequence is provided in Fig. 4 .

**Fig. 3:**
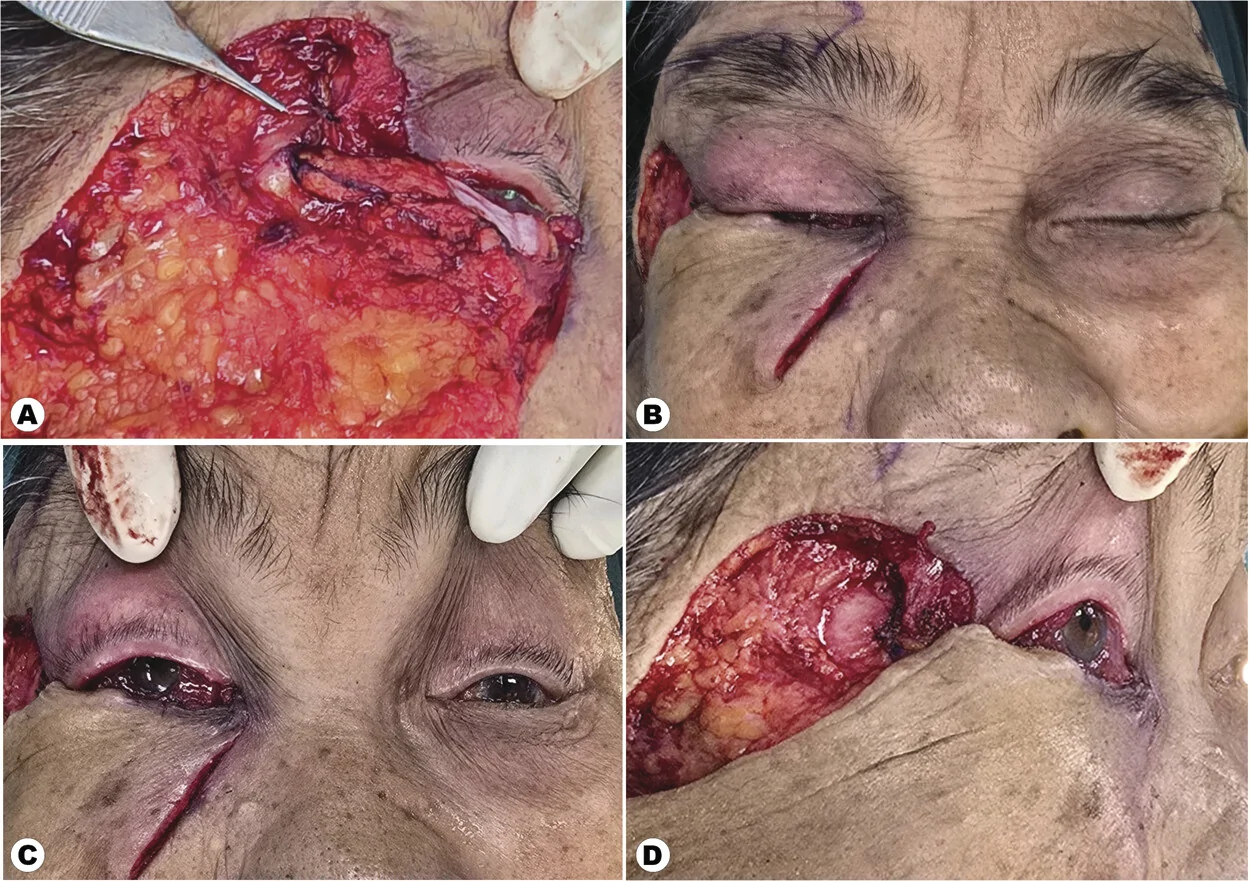
Intraoperative photographs demonstrating completed reconstruction. ( **A** ) Primary donor site closure. ( **B–D** ) Completed reconstruction with preserved lower eyelid position.

**Fig. 4:**
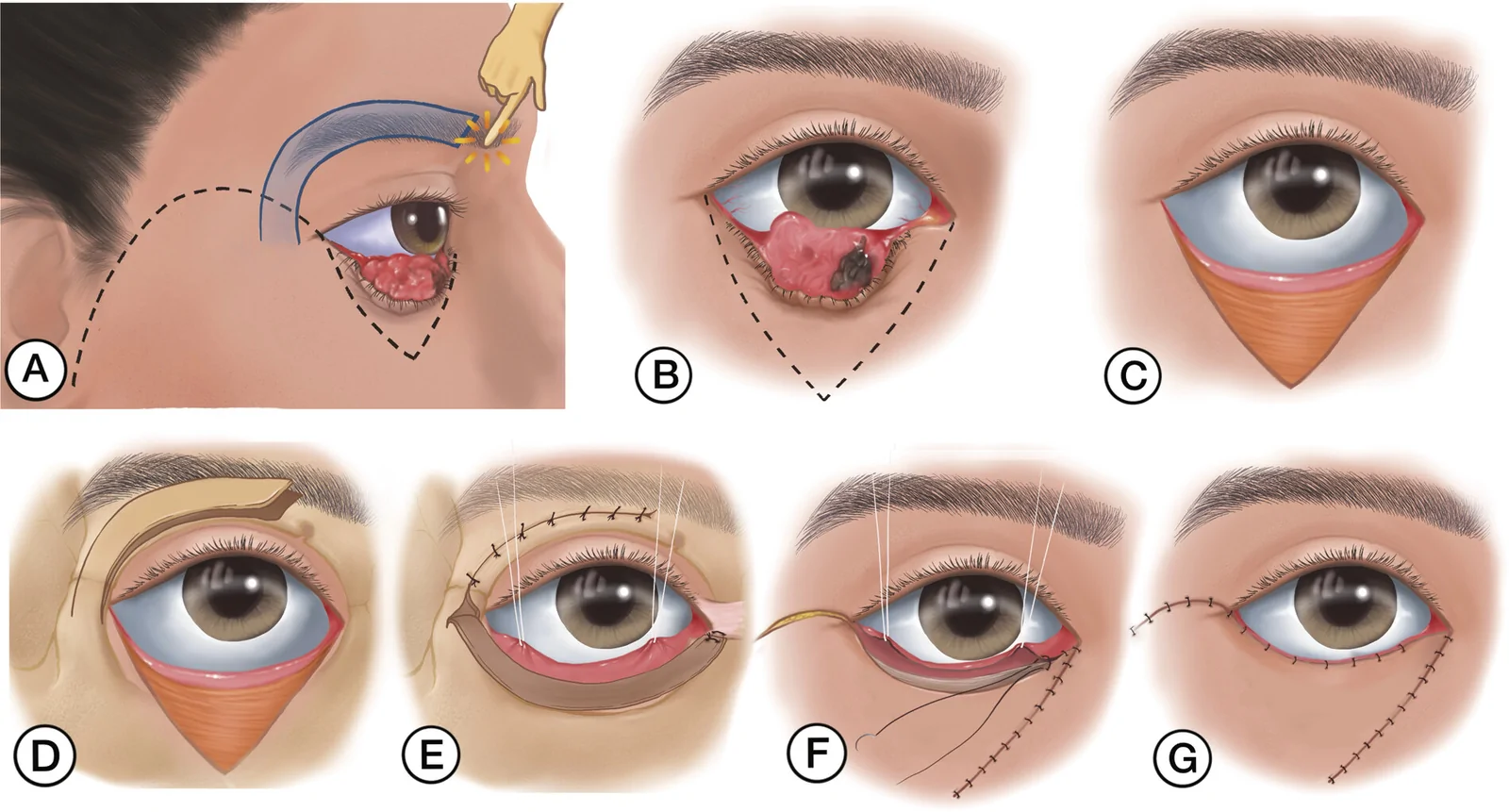
Schematic illustration of the extended superolateral periosteal flap technique. ( **A** ) Lateral view of preoperative planning. Black dashed lines outline the 5-mm oncologic excision margin around the lower-eyelid lesion and the planned cheek rotation flap; blue solid lines indicate the planned superolateral periosteal flap (35 mm × 6–8 mm) along the superior orbital rim; the fingertip marks the palpated supraorbital notch, which serves as the medial boundary for safe dissection. ( **B** ) Frontal view of preoperative planning, showing the lower-eyelid lesion with the 5-mm oncologic excision margin (black dashed line). ( **C** ) Postexcisional full-thickness total lower-eyelid defect. ( **D** ) Subperiosteal elevation of the periosteal flap is performed from medial to lateral: Dissection begins at the medial endpoint within 2 to 3 mm of the supraorbital notch, extends along the superior orbital rim across the frontozygomatic suture, and stops at the lateral canthal region, where the lateral canthal attachment is preserved as the vascular pedicle. ( **E** ) Inferior transposition of the flap with primary closure of the brow donor site along the superior orbital rim using 4–0 absorbable sutures, tension-free fixation of the distal flap end to the medial canthal tendon remnant using a 6–0 non-absorbable monofilament suture in a horizontal mattress configuration, and advancement of the inferior fornix conjunctiva secured by temporary unknotted hanging sutures. ( **F** ) Cheek rotation flap elevated and advanced medially to cover the anterior lamella; the periosteum, conjunctiva, and intradermal skin layers are secured in three layers with 5–0 absorbable sutures using a buried suture technique. ( **G** ) Final reconstruction with primary closure of the cheek donor site.

At 2-week follow-up, the reconstructed lower eyelid demonstrated satisfactory eyelid position with expected postoperative chemosis and mild upper eyelid edema ( Fig. 5A, B ). At 3-month follow-up, a stable margin position with preserved alignment and complete flap survival was achieved. No ectropion, entropion, symblepharon, lagophthalmos, or flap necrosis was observed ( Fig. 5C, D ). Chemosis and upper eyelid edema resolved completely. Persistent epiphora was attributed to oncologic resection of the lower lacrimal drainage system. Longer-term follow-up was limited due to patient's death from an unrelated medical condition.

**Fig. 5:**
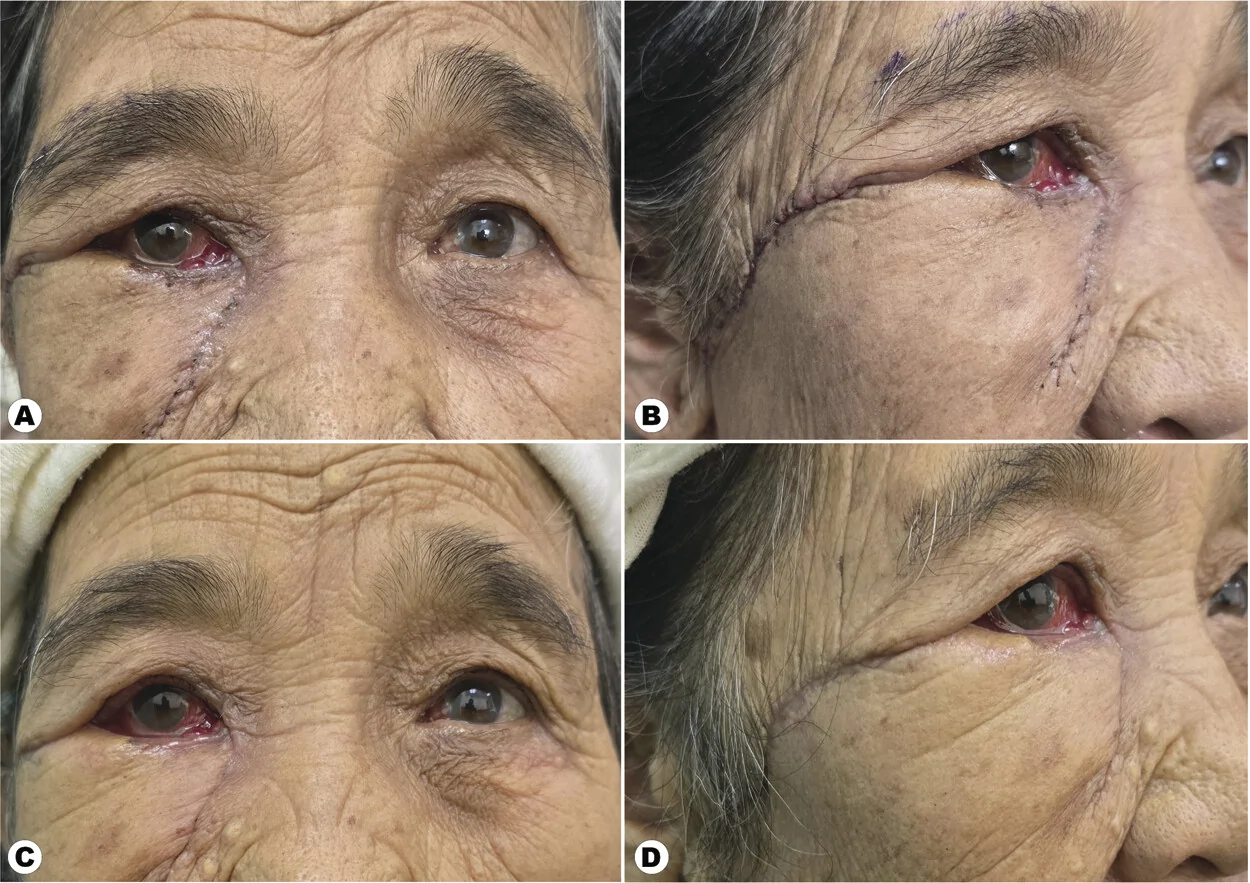
Postoperative photographs at ( **A, B** ) 2 weeks and ( **C, D** ) 3 months. ( **A** ) Frontal view at 2 weeks demonstrating satisfactory eyelid position with postoperative chemosis. ( **B** ) Lateral view at 2 weeks demonstrating early scar maturation. ( **C** ) Frontal view at 3 months demonstrating stable eyelid margin position with complete resolution of chemosis. ( **D** ) Lateral view at 3 months demonstrating mature scar and satisfactory contour.

## Discussion

In total full-thickness lower-eyelid reconstruction, the choice of posterior lamellar substitute is a principal determinant of long-term margin position and resistance to ectropion. The 35-mm flap length extends the achievable length of previously described periosteal flap techniques, including a previously reported 15- to 20-mm technique 4 and Kono et al's 25-mm L-shaped configuration, 5 enabling tension-free medial canthal fixation and potentially extending reconstruction of total defects beyond the traditionally reported limits of periosteal flaps. Dissection along the superior orbital rim may reduce the risk of injury to the frontal branch of the facial nerve.

This extended periosteal flap technique may be particularly useful for large full-thickness lower eyelid defects when single-stage reconstruction is preferred and prolonged eyelid occlusion is undesirable. It is especially applicable when adequate superior orbital rim periosteum is preserved, and local vascularity is reliable. The method may be less suitable in patients with prior superior orbital rim surgery, extensive periosteal scarring, or severe local radiation damage that could compromise flap vascularity. A history of frontal or zygomaticofrontal fracture with prior internal fixation is also a relative contraindication, as previous subperiosteal stripping or osteosynthesis hardware at the donor site may have devitalized or disrupted the periosteum.

Unlike acellular dermal matrices requiring 3 to 6 months of eyelid fusion, 2 3 the vascularized periosteal flap permits single-stage completion, preserving binocular vision. Conjunctival advancement prevents symblepharon formation. Free tarsoconjunctival grafts from the contralateral upper eyelid more closely reproduce native anatomy and provide intrinsic rigidity that may offer greater long-term resistance to vertical shortening or late entropion. 8 9 10 Stephenson and Brown 8 reported successful use of autogenous tarsal grafts in 121 procedures with no graft failures; Leone and Van Gemert 9 combined free tarsoconjunctival grafts with bipedicle skin muscle flaps for single-stage reconstruction; and Bortz and Al-Shweiki 10 demonstrated acceptable results, although 50% required secondary revision. However, graft harvesting must preserve at least 4 mm of upper tarsal height, which restricts graft dimensions, 8 and free grafts depend entirely on recipient bed perfusion.

An additional posterior lamellar option is the hard palate mucoperiosteum; a recent series combining a hard palate graft with a Fricke flap reported satisfactory single-stage reconstruction of extensive lower-eyelid defects in elderly patients with low morbidity, although the graft remains dependent on recipient bed perfusion and adds palatal donor site morbidity. 11 For the anterior lamella, established options include the Tenzel semicircular rotation flap for moderate defects, 12 13 the cheek rotation (Mustardé) flap for larger defects, and the Fricke or paramedian forehead flap for very extensive defects, particularly after failed prior repair or radiation injury, although forehead-based reconstructions are typically multistage and leave a forehead donor scar. 14 15 In the present case, a cheek rotation flap was selected for the anterior lamella, while the extended superolateral periosteal flap provided an intrinsically vascularized posterior lamellar layer in a single stage.

The principal advantages of the technique are the vascularized pedicle, which ensures reliable flap survival independent of recipient bed perfusion and is particularly useful in previously operated or irradiated fields, the 35-mm flap length exceeding achievable tarsal graft dimensions, and the avoidance of contralateral upper-eyelid donor morbidity. Although periosteum does not possess the intrinsic rigidity of native tarsus, functional posterior lamellar support depends not only on material stiffness but also on fixation geometry and canthal anchoring. In this technique, combined medial and lateral periosteal fixation with conjunctival lining provided sufficient early structural stability. Short-term clinical behavior suggests that properly tensioned periosteal support can maintain margin position in selected cases. Longer follow-up is nevertheless required to determine resistance to late cicatricial contraction and progressive vertical shortening.

Several technical considerations merit emphasis. A true subperiosteal dissection plane must be maintained to preserve flap integrity and vascularity, and the medial extent of dissection should stop several millimeters lateral to the supraorbital notch to reduce neurovascular injury risk. Excessive thinning should be avoided, as adequate flap thickness contributes to mechanical durability during eyelid movement, and flap length should be planned according to defect width and the required canthal fixation distance. Tension-free medial fixation is critical because excessive traction can produce early vertical shortening or margin malposition. Inferior transposition along a gentle curvature rather than a sharp angulation helps maintain pedicle perfusion, and conjunctival advancement should fully cover the periosteal surface to minimize adhesion and symblepharon formation.

This case has certain limitations. First, the findings are derived from a single patient, and further validation in a larger series of patients is required before generalization. Second, follow-up was limited to 3 months because of the patient's death from an unrelated medical condition. Despite the truncated follow-up period, the results demonstrated stable eyelid position, complete flap survival, and satisfactory functional outcomes. Longer-term assessment of late cicatricial changes or progressive retraction was not possible. This technique provides adequate posterior lamellar support for single-stage reconstruction of total lower eyelid defects, although further cases are required to assess reproducibility across different patient populations and long-term outcomes.
